# Two paralogous *znf143* genes in zebrafish encode transcriptional activator proteins with similar functions but expressed at different levels during early development

**DOI:** 10.1186/s12860-020-0247-7

**Published:** 2020-01-22

**Authors:** Laura Huning, Gary R. Kunkel

**Affiliations:** Department of Biochemistry and Biophysics, Texas A&M University, College Station, TX 77843-2128 USA

**Keywords:** Eukaryotic transcription, Transcription factor, Zebrafish, Paralogous genes, ZNF143

## Abstract

**Background:**

ZNF143 is an important transcriptional regulator protein conserved in metazoans and estimated to bind over 2000 promoter regions of both messenger RNA and small nuclear RNA genes. The use of zebrafish is a useful model system to study vertebrate gene expression and development. Here we characterize *znf143a*, a novel paralog of *znf143b*, previously known simply as *znf143* in zebrafish. This study reveals a comparison of quantitative and spatial expression patterns, transcriptional activity, and a knockdown analysis of both ZNF143 proteins.

**Results:**

ZNF143a and ZNF143b have a fairly strong conservation with 65% amino acid sequence identity, and both are potent activators in transient transfection experiments. In situ hybridization analyses of both *znf143* mRNAs show that these genes are expressed strongly in regions of the brain at 24 h post fertilization in zebrafish development. A transient knockdown analysis of *znf143* expression from either gene using CRISPR interference revealed similar morphological defects in brain development, and caused brain abnormalities in up to 50% of injected embryos. Although present in the same tissues, *znf143a* is expressed at a higher level in early development which might confer an evolutionary benefit for the maintenance of two paralogs in zebrafish.

**Conclusions:**

*znf143a* encodes a strong activator protein with high expression in neural tissues during early embryogenesis in zebrafish. Similar to its paralogous gene, *znf143b*, both *znf143* genes are required for normal development in zebrafish.

## Background

Zinc Finger Protein 143 (ZNF143) is a sequence-specific transcriptional activator protein involved in stimulation of transcription from over 2000 mammalian promoters [1, 2]. ZNF143 binds to so-called *Sph*I Postoctamer Homology (SPH) motifs or Staf (Selenocysteine transcription activating factor) Binding Sites (SBSs) that are located typically within a couple hundred base pairs of the transcriptional start site of gene promoters. ZNF143 was characterized initially for its transcriptional activation activity and more recently for its occupancy at the boundaries of topologically associated domains in chromatin along with the CCCTC-Binding Factor (CTCF) protein [3–10]. ZNF143 is able to regulate transcription from both small nuclear RNA (snRNA) promoters and mRNA promoters by RNA polymerase II or III [6]. Additionally, ZNF143 has been implicated in regulation of cell cycle progression and tumor growth [2, 11–15], and is an important regulator involved in zebrafish development [16]. However, past functional studies of ZNF143 in zebrafish have not considered a previously uncharacterized paralogous gene (*znf143a*) also expressed during early development.

Gene duplication is thought to bestow a long-term evolutionary advantage due to a decreased number of constraints present on one of the functional copies of the gene [17]. Zebrafish, among other teleost fish, have an increased copy number of genes due to a third teleost specific whole genome duplication event occurring 100 million years ago [18]. Therefore, an additional copy of the gene could incorporate mutations that may confer novel function or expression. The previously studied gene, *znf143b,* has an uncharacterized paralog located on chromosome 7. Here we investigate this paralog, *znf143a*, including its quantitative and spatial expression patterns, its ability to activate mRNA promoters, and comparative phenotypic knockdown analysis through the use of CRISPR interference (CRISPRi).

## Results

**Fig. 1:**
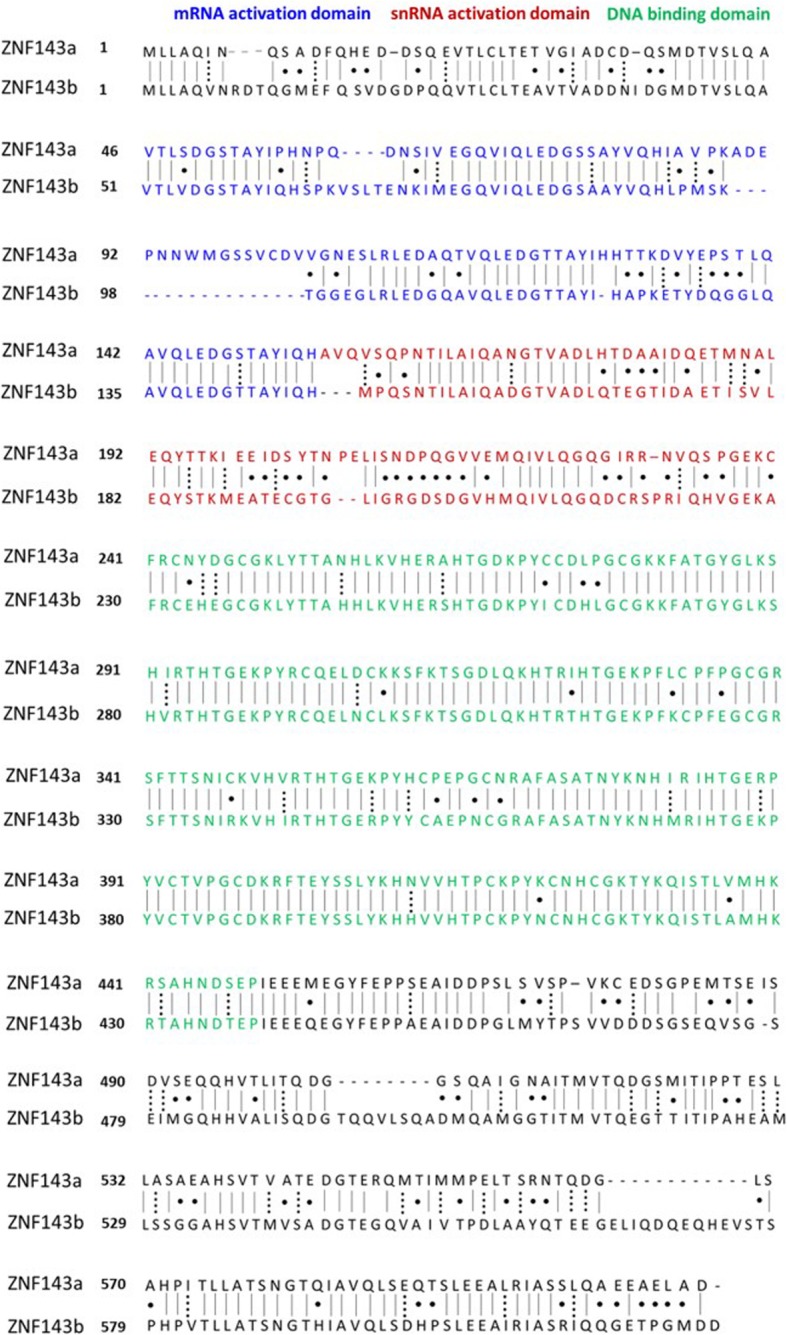
Amino acid sequence alignment of zebrafish ZNF143a vs. ZNF143b. Protein sequences of ZNF143a and ZNF143b were aligned with the *lalign* sequence analysis program using the algorithm of Huang and Miller [19]. Identical amino acids are indicated by a straight line, similar amino acids are indicated by a dotted line, and dissimilar amino acids are indicated by a single dot. Amino acids within the DNA binding domain are colored green, amino acids within the mRNA activation region are colored blue, and amino acids within the snRNA gene activation region are colored red

We characterized the novel gene *znf143a*, a paralog of the previously studied *znf143b* (protein also named Staf or SPH Binding Factor (SBF)). Quantitative expression levels, spatial expression patterns, transcriptional activation potentials of the encoded proteins, and phenotypic analyses following knockdown of both genes are reported. The two ZNF143 proteins exhibit a large sequence homology containing 65% amino acid identity, with most of this conservation in the DNA binding domain (DBD) residing in the seven C2H2 zinc fingers located between amino acids 229–438 (Fig. 1). ZNF143a encodes a slightly shorter protein containing 613 amino acids instead of the 623 amino acid ZNF143b protein. Deviations in sequence identity between the two proteins primarily exist within the amino-terminal region, containing the mRNA activation domain from residues 51–149 (ZNF143b) and the snRNA activation domain from 150 to 228 (ZNF143b), and the carboxy-terminal region of unknown function (Fig. 1).

**Fig. 2:**
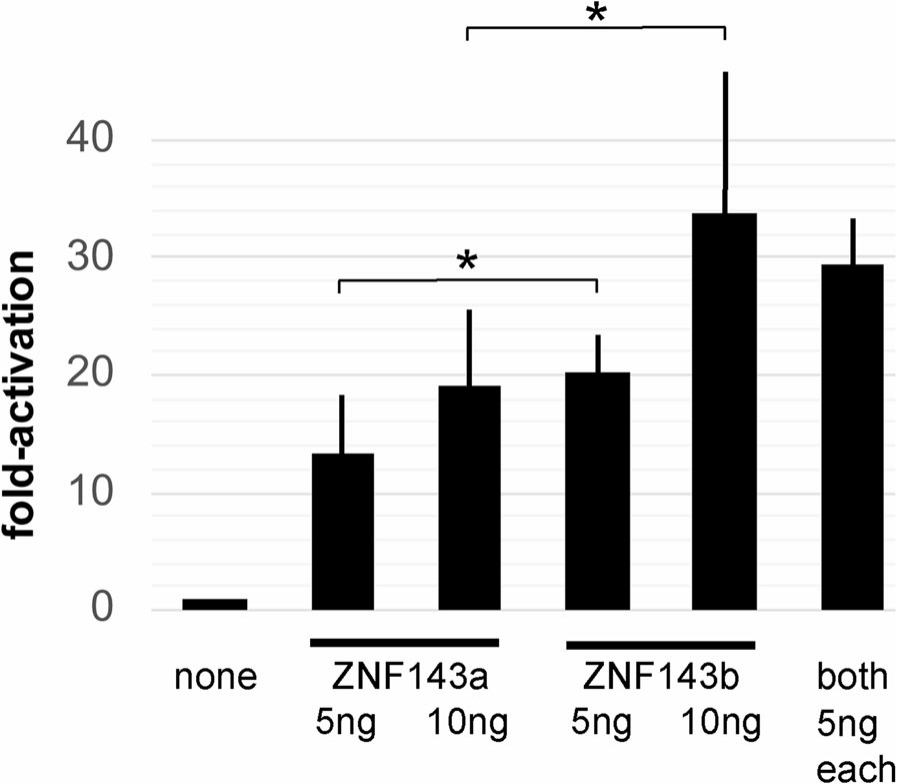
ZNF143a and ZNF143b exhibit similar transcriptional activation potential. Zebrafish ZF4 cells were transfected with pGL3-SPH5 firefly luciferase reporter gene plasmid, plus pRL-SV40 renilla luciferase reporter plasmid, and as noted, pCI-myczznf143a or pCI-myczznf143b expression vector plasmid. Relative luciferase expression was determined by comparing the firefly/renilla luciferase ratio for each sample to that ratio for the sample with addition of expression vector plasmid containing no gene. Bar height shows the mean value from independently transfected wells, and error bars report the standard deviation from the mean. The single asterisk denotes a significant difference (*p* < 0.05). As noted in a previous publication [16], we were unable to detect expression of the myc-tagged ZNF143 in ZF4 cells. Expression of ZNF143b in human HEK293 cells was somewhat higher than ZNF143a, and if representative of relative synthesis in ZF4 cells, could explain the greater transcriptional activation by ZNF143b in transient transfection experiments

Due to some sequence differences in the activation domain regions of the N-terminal region of the protein, we hypothesized that the transcriptional activation properties between the two proteins may differ. It has been demonstrated that zebrafish ZNF143b can bind and activate mRNA promoter regions such as the *pax2a* promoter [16]. To assess the capacity of ZNF143a to act as a transcriptional activator protein at mRNA promoters, transient transfection assays were performed using cultured ZF4 zebrafish cells. A synthetic promoter containing five binding sites for ZNF143 was used to drive a luciferase reporter. The addition of *znf143a* expression vector plasmid at 5 ng and 10 ng led to a significant increase of transcription, 13–19 times the amount of luciferase activity relative to the empty expression vector control (Fig. 2). Furthermore, we did not detect any synergistic effect after adding 5 ng of each *znf143* expression plasmid. We note that previous work demonstrated a direct effect of ZNF143b in such experiments, dependent on the presence of SPH sites in the promoter and the activation domains in ZNF143b [16]. Although there was a significant difference in activation levels between ZNF143a and ZNF143b (Fig. 2), their relative potencies were unable to be compared quantitatively due to possible differential protein expression, as noticed in human embryonic kidney (HEK293) cells (results not shown). Similar to earlier work [16], we were unable to detect myc-tagged ZNF143 in transfected zebrafish cells. Nevertheless, ZNF143a can act as a strong transcriptional activator protein.

**Fig. 3:**
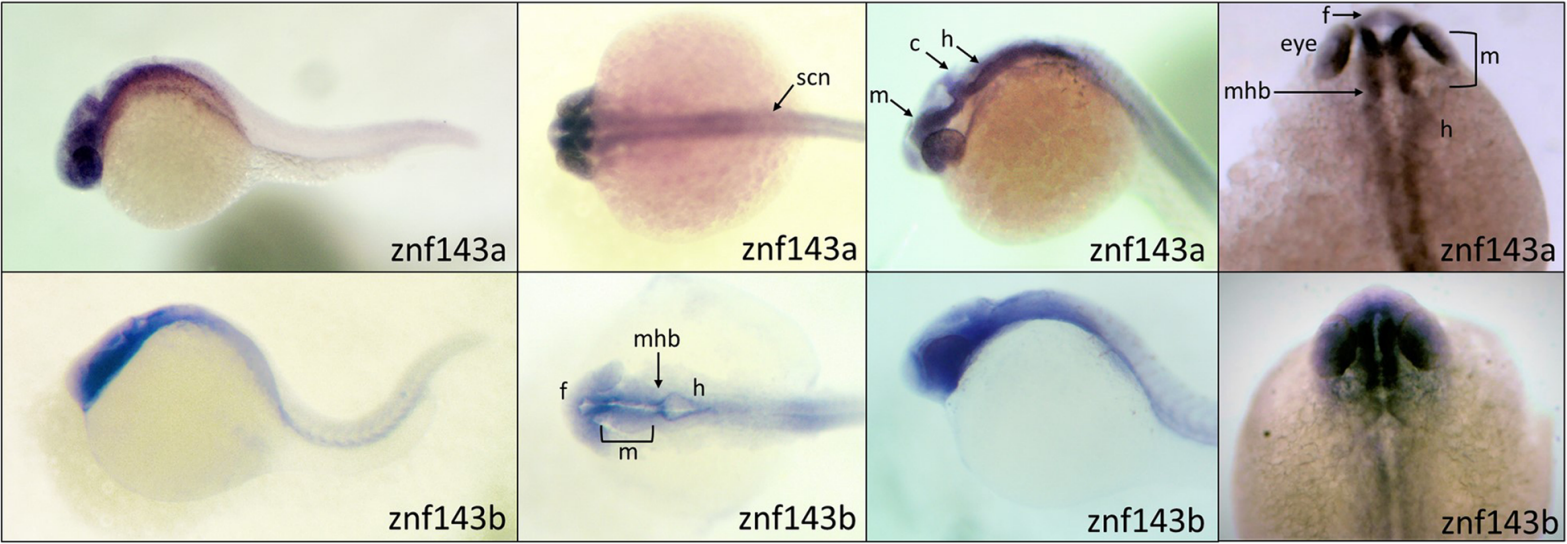
Similar spatial expression of *znf143a* and *znf143b* in 24hpf embryos. Antisense digoxigenin riboprobes against the *znf143a* and *znf143b* were used for whole-mount in situ hybridization assays. The probe for *znf143b* targeted the last exon of the coding region (approximately 186 nt corresponding to the last 61 amino acids) and the 3’UTR of the gene, while the probe for *znf143a* was designed solely against the 3’UTR. Embryos used were fixed and stained at 24hpf. The panels illustrate views from four different embryos for each probe, with the right-most panels showing an enlarged dorsal view of the head. We note that in all experiments, the *znf143b* probe produced fainter staining, most likely because of a smaller than optimal riboprobe that was necessary to ensure gene specificity. Specific head, brain and neural structures are identified with the following abbreviations: f, forebrain; m, midbrain; h, hindbrain; mhb, midbrain hindbrain boundary; c, cerebellum; scn, spinal cord neurons

Gene duplications in vertebrates can lead to a divergence in tissue specific expression that may be suggestive of varying contribution of paralogous genes to specific organ functions [17]. We hypothesized that *znf143a* may be expressed in a tissue-type distinct from *znf143b*. Tissue specific expression patterns of both of the *znf143* mRNAs were determined by in situ hybridization analyses using digoxigenin (DIG)-labeled antisense riboprobes. Due to a high sequence similarity of the two coding sequences, antisense probes were designed to target primarily the 3′-untranslated region (3’UTR) sequences of each *znf143* paralog. Both riboprobes for the paralog genes demonstrated strong expression in the brain in 24 h post fertilization (hpf) zebrafish embryos including the forebrain, midbrain, and hindbrain regions (Fig. 3). As evident from similar hybridization patterns, expression of *znf143a* and *znf143b* genes are not spatially distinct.

**Fig. 4:**
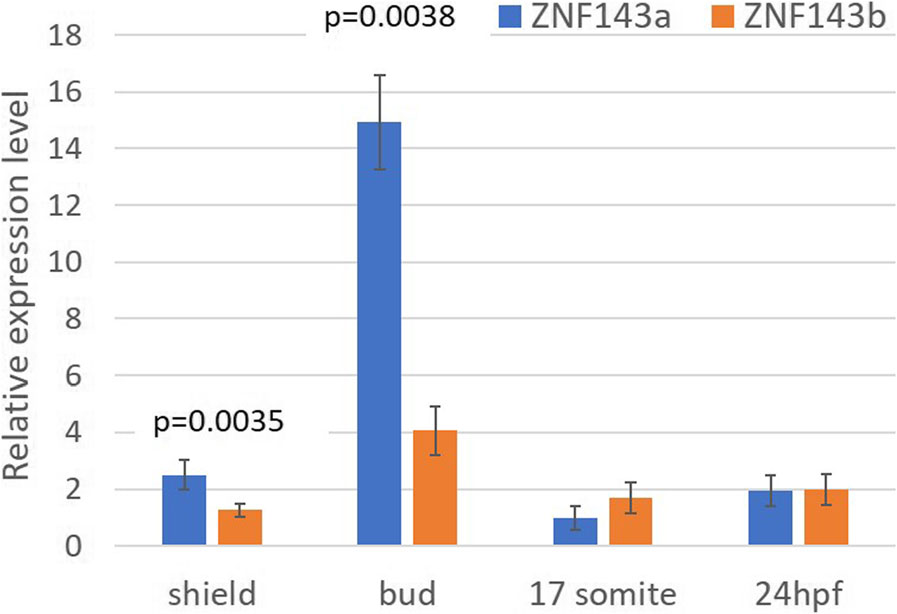
*znf143* paralog genes are differentially expressed in early development in zebrafish. cDNA was obtained from total RNA isolated from zebrafish embryos at either the shield, bud, 17-somite, or 24hpf stages. qPCR was performed for both *znf143a* and *znf143b.* Numbers on the y-axis represent the mean values for relative expression of each gene after normalization to the geometric mean of two housekeeping gene controls (*ef1α* and *rpl13α*), and comparison to the lowest value (*znf143a* at 17-somite stage). Error bars represent standard deviation from the mean. Statistically significant differences in expression levels are signified by the inclusion of *p*-values< 0.05, determined by Student’s t test

Another possible outcome with two different *znf143* genes is that they may be expressed at a different developmental time periods during zebrafish embryogenesis. To explore this hypothesis, total RNA from embryos was isolated at various developmental time points including shield (6hpf), bud (10hpf), 17-somite (16hpf), and 24hpf, and converted to complementary DNA (cDNA). Quantitative PCR analyses showed that *znf143a* is expressed significantly higher in early development especially at the shield and bud stages, while *znf143b* is expressed at similar expression levels in the first 24 h of development (Fig. 4). Both *znf143a* and *znf143b* are expressed at a similar level by 24hpf. The expression difference between bud stage and 24hpf embryos was verified by using additional primer pairs for each gene (results not shown). In these latter qRT-PCR experiments the expression level of *znf143a* decreased 2.5-fold between bud and 24hpf, while the level of *znf143b* remained constant.

**Fig. 5:**
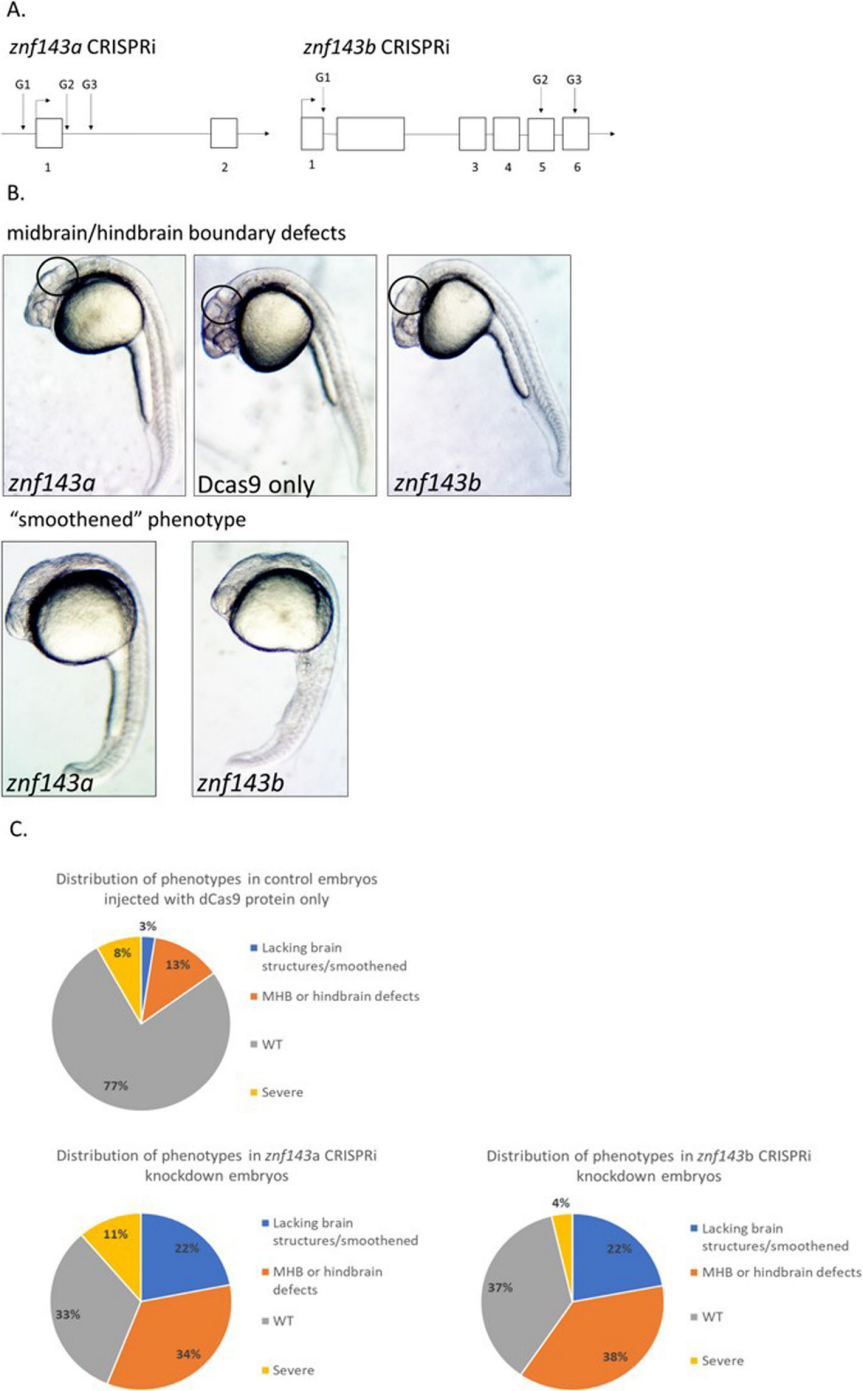
CRISPRi knockdown of either *znf143a* or *znf143b* induce brain developmental defects. **a**. Diagram showing targets for sgRNAs. Numbered boxes indicate positions of exons. **b**. One-cell zebrafish embryos were injected with sgRNA/dCas9 protein complexes to knock down *znf143a*, *znf143b*, or in control experiments with dCas9 protein only (−sgRNA) and observed at 24hpf. Representative photographs of abnormal brain phenotypes are shown, with circled regions showing the areas of most interest. **c**. Consistent phenotypes are grouped into classes. Severe phenotypes lacked axial development. 159 embryos were counted for the control injections lacking any sgRNAs, 132 embryos were counted for the *znf143a* CRISPRi injections, and 136 embryos were counted for the *znf143b* CRISPRi injections

**Fig. 6:**
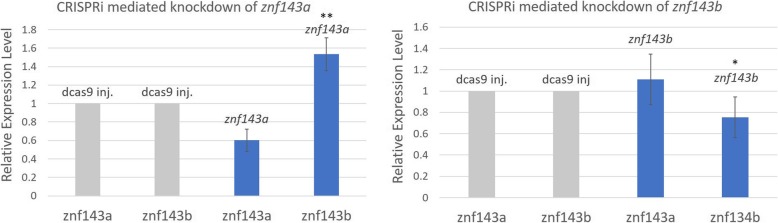
Analysis of specific gene knockdown in CRISPRi experiments. Quantitative RT-PCR was used to analyze relative levels of *znf143a* and *znf143b* transcripts. The amounts of each *znf143* cDNA were determined relative to those in the dCas9 injection control after normalization to the geometric mean of the widely-expressed transcripts (*ef1α* and *rpl13α*). The height of each column represents the mean of 4 or 5 independent injection experiments, and error bars represent standard deviation from the mean. A single asterisk signifies a *p*-value < 0.05 relative to the control sample lacking any sgRNAs, while a double asterisk signifies a *p*-value < 0.01

The advent of CRISPR technologies has added a new method to knock down gene function. Repression through CRISPR/Cas9 uses a guide RNA-directed deactivated Cas9 (dCas9) protein as a roadblock for transcriptional initiation or elongation, known as CRISPRi [20, 21]. This method has been applied to study transient gene knockdown in both *C. elegans* and zebrafish that resulted in mild morphological phenotypes [22]. In this earlier report the injection of multiple strand specific guide RNAs improved gene repression using CRISPRi [22]. We used this CRISPRi method to study individual gene function of either *znf143a* or *znf143b*. Three single guide RNAs (sgRNAs) were designed to target the early transcribed regions of the *znf143a* or *znf143b* genes (Fig. 5a). Embryos displayed similar phenotypes when knockdown was elicited with three sgRNAs targeting *znf143a* or *znf143b* (Fig. 5b,c). Phenotypic defects included a loss of midbrain/hindbrain formation, hindbrain enlargement, and an overall loss of brain organization. Knockdown of either paralog resulted in a complete loss of brain formation in about 22% of injected embryos (Fig. 5c). These fish are referred to as a “smoothened” phenotype due to a lack of brain structures causing a uniform unwrinkled head. Despite such massive defects in the brain, the “smoothened” fish still underwent somitogenesis and displayed a developed axis. A low percentage of injected knockdown embryos (less than 11%) displayed severe defects including a shortened axis (Fig. 5c). Embryos injected with either set of gRNAs, but no dCas9 protein, did not display any phenotypic effects (results not shown). A small proportion of embryos injected only with dCas9 protein displayed abnormal phenotypes (Fig. 5c). Therefore, knocking down a single paralog, either *znf143a* or *znf143b*, was sufficient to exhibit brain defects. Although these genes have redundant functions, both are essential to zebrafish development. The specificity of gene-specific knockdown was investigated in quantitative reverse transcriptase polymerase chain reaction (qRT-PCR) experiments that demonstrated modest, but significant reduction in mRNA levels (25–40%) (Fig. 6). The moderate reductions in mRNA levels observed are similar in magnitude to other reported multiple guide CRISPRi knockdowns in zebrafish [22]. Interestingly, knockdown of *znf143a* caused a 1.5-fold increase in total *znf143b* mRNA, an effect that was not apparent with *znf143b* knockdown (Fig. 6).

## Discussion

In this study, we show that two versions of zebrafish ZNF143 expressed from paralogous genes have similar functions, including strong transcriptional activation potential, comparable spatial expression during embryogenesis, and identical phenotypic outcomes after partial knockdown. The only notable difference that we detected was relatively higher expression of *znf143a* during early zebrafish development. Although global roles for both ZNF143a and ZNF143b are suggested by phenotypic effects following individual knockdown, future RNAseq experiments could illustrate the scope of paralog specific regulation.

Genome duplications can lead to gene paralogs that evolve different functions or tissue specificities over time. However, this is not the case with *znf143a* and *znf143b* as both exhibited similar spatial expression patterns and likely perform similar tissue-specific roles. Both proteins act as potent activators in the cell, but we did not observe a synergistic activation in transfection assays. Each paralog gene encodes for a protein able to stimulate transcription from target promoters through SPH sites. Despite conserved expression patterns, both paralogs appear to be important for zebrafish development when targeted individually for CRISPRi knockdown analyses. We did not observe any exacerbation of phenotypic effects when expression from both paralogs was reduced simultaneously (data not shown).

In humans, there are also two *znf143* paralog genes, *znf143* and *znf76* [23]*.* Both genes are highly conserved among mammals. Human ZNF76 contains 57% amino acid sequence identity and 78% similarity to human ZNF143. Both proteins are able to bind and activate promoters of genes transcribed by RNA polymerase II or RNA polymerase III containing SPH sites, and are highly expressed in most tissues [23]. Zebrafish also contain the conserved *znf76* paralog. The extra *znf143* paralog, *znf143a*, is only retained in teleost fish.

The promoters for both zebrafish *znf143* paralogs contain a putative SPH binding site suggesting a potential autoregulatory feedback loop at both genes. Human ZNF143 and ZNF76, are able to bind to SPH sites with similar affinities and activate the *znf143* promoter [1]. When *znf143* or *znf76* is over-expressed in cells, this can downregulate the levels of endogenous ZNF143 [24]. When ZNF143 is saturated in the cell, it has the ability to bind to non-canonical binding sites, and this produces a transcriptional start site switch mechanism that can result in two different transcripts with one transcript much more efficient at production of a protein [24]. It is possible that ZNF143a, due to its ability to bind and activate SPH sites, also contributes to a similar auto-regulatory feedback loop that regulates the *znf143* gene. Alternatively, ZNF143b may be able to bind and regulate expression of *znf143a* due to the presence of an SPH site in its promoter. This cross-talk between *znf143* and other *znf143* paralogs may contribute to the fitness of the zebrafish and be an important factor when knocking down a single paralog as witnessed in knockdown experiments. The existence of ZNF143a may be another regulating factor crucial for maintaining the correct expression levels of ZNF143b.

There are multiple possibilities as to why both *znf143a* and *znf143b* are critical for the early development of zebrafish. One possibility is that *znf143a* encodes for a protein that confers a function that is different from *znf143b*. Although zebrafish ZNF143 proteins contain 65% sequence identity, there are a few regions with divergent sequence. One such region is C-terminal to the DBD, and accounts for unknown function in both ZNF143 paralogs. ZNF143a contains two deletions in the C-terminal region of the protein encompassing 8 amino acids and 12 amino acids when compared to ZNF143b (Fig. 1). A second possibility for the requirement of both *znf143* paralogs for healthy development is the temporal difference in quantitative expression of *znf143a* and *znf143b*. Coordinated control of gene expression, both spatially and temporally, is critical for proper development of an organism. Expression of *znf143a* early in development or later expression of *znf143b* could help maintain the specific chromatin environment necessary for the correct gene products to be produced at the correct time during development of the zebrafish embryo. Lastly, paralogous duplicate genes may be retained in evolution to allow more control of gene dosage [25, 26]. Multiple copies of *znf143* that express at different times could contribute to a more finely-tuned dosage effect that contributes to the overall fitness of the zebrafish during development. Necessity for a narrow window of *znf143a* or *znf143b* gene dosage could provide a rationale for the strong phenotypic defects observed in our CRISPRi knockdowns, despite only having a modest decrease in overall quantitative gene expression. In addition, paralogous versions of *znf143* can buffer mutations arising in either gene by the mechanism known as transcriptional adaptation [26, 27]. Possibly, the modest increase in *znf143b* mRNA level after CRISPRi knockdown of *znf143a* (Fig. 6) could reflect transcriptional adaptation induced by short transcripts produced by knockdown with guide RNAs binding downstream of the transcriptional start site.

## Conclusions

The identification and delineation of paralogous genes is critical for understanding gene function. We have characterized a novel paralog of a pervasive eukaryotic transcriptional activator protein encoded by the gene *znf143b*, called *znf143a* in zebrafish. In addition to ZNF143b, ZNF143a is a potent transcriptional activator protein capable of activating protein coding genes as shown by transient transfection assays. We have demonstrated that both *znf143a* and *znf143b* are expressed in the brain in 24hpf zebrafish embryos. Though displaying similar tissue-specific expression patterns, *znf143a* is expressed quantitatively at higher levels earlier in development than *znf143b*. Both paralogs exhibited developmental defects in the brain when targeted for knockdown using CRISPRi, and in severe cases those defects were seen throughout the axis of the embryo.

## Methods

### Plasmid constructions

The pCI-myczznf143b expression plasmid and pGL3-SPH5 firefly luciferase reporter plasmid were described previously [16]. In order to construct the pCI-myczznf143a plasmid, three fragments of *znf143a* were amplified by PCR from zebrafish cDNA and ligated together using naturally occurring restriction enzymes within the *znf143a* sequence and an engineered *Mlu*I site and *Not*I site added into the 5′-end and 3′-end primers, respectively. Additionally, a single myc tag was engineered at the amino-terminus. The first and second fragments were ligated using a naturally occurring unique *Eco*RI site, and the second and third fragments joined using a naturally occurring unique *Bam*HI site. The PCR fragments were restricted with the appropriate restriction enzymes, gel-purified, and ligated together into the pCI-neo vector (Promega) that was previously restricted with *Mlu*I and *Not*I. The *znf143a* sequence was verified by the dideoxy method.

A plasmid was constructed using the pGEM-T vector (Promega) in order to synthesize a *znf143a* riboprobe for in situ hybridization assays. pGEM-znf143a was designed to target the 3’UTR of *znf143a*, as this region of the gene was the most distinct from *znf143b*. Primers used to amplify the 3’UTR region of *znf143a* had the following sequences: 5′- CCACCTTCACCTTGAGAC-3′, and 5′- AATATCACCATCATCAGTTTA-3′.

Plasmids were constructed using the pDR274 vector [28], obtained from AddGene, in order to synthesize single guide RNAs (sgRNAs) for CRISPRi. Guide RNAs were designed stepwise in the sense direction to target the 5′ transcribed regions of either *znf143b* or *znf143a*, and construction of the pDR274 plasmids followed a standard protocol [28]. Guide RNAs targeted the first intronic region of the *znf143a* gene with the following primers: Guide 1 Forward: 5′-TAGGCGATCTGCAGTACGTTACA-3′, Reverse: 5′-AAACTGTAACGTACTGCAGATCG-3′; Guide 2 Forward: 5′-TAGGTGAAACTAGATATCGCTGC-3′, Reverse: 5′-AAACGCAGCGATATCTAGTTTCA-3′; Guide 3 Forward: 5′-TAGGAAACTAACGTTACACGCCT-3′, Reverse: 5′-AAACAGGCGTGTAACGTTAGTTT-3′. Guides targeted the first exon of the *znf143b* gene with the following primers: Guide 1 Forward: 5′-TAGGTGCATGGTGGTCGAACGA-3′, Reverse: 5′-AAACTCGTTCGACCACCATGCA-3′; Guide 2 Forward: 5′-TAGGGCATGGAGTTTCAGAGTG-3′, Reverse: 5′-AAACCACTCTGAAACTCCATGC-3′; Guide 3 Forward: 5′-TAGGACAAGTGATTCAGCTGG-3′, Reverse: 5′-AAACCCAGCTGAATCACTTGTC-3′. Oligonucleotides were annealed and ligated into the *Bsa*I site of pDR274 [28]. Sequences were verified using the dideoxy method.

### Cell culture, transfection, and reporter gene assays

Transient transfection experiments in zebrafish ZF4 cells or human HEK293 cells were performed as described previously [16]. Protocols with HEK293 cells followed BSL-2 guidelines approved by the Texas A&M Institutional Biosafety Committee (Permit IBC2016–047).

### RNA isolation, cDNA synthesis, RT-PCR

Total RNA was isolated from wild-type zebrafish embryos at designated developmental stages, converted to cDNA, and used in qRT-PCR experiments as described previously [29]. Gene-specific primers for *znf143a* were: 5′-GCGGTTCCAAAAGCAGATGAGC-3′ and 5′-CTTCCAGCTGAACGGTCTGAGC-3′, and for *znf143b* were: 5′-CTCTACTAAGATGGAGGCCACAG-3′ and 5′-CTGGATTCTAGGTGAACGACAGTC-3′ These primer sets were chosen for binding to nonhomologous regions of the two open reading frames (ORFs).

### Zebrafish husbandry, and in situ hybridization

AB/TL wild-type strain zebrafish for breeding to produce embryos were obtained from colleagues in the Biology Department, Texas A&M University, and were maintained using standard methods with protocols approved by the Texas A&M University Animal Care and Use Committee (AUP #2016–0102 and AUP #2019–0139). After use for embryo production, adult fish were returned to breeding stock tanks containing 10–20 animals. Embryos used for in situ hybridization or injection experiments were chosen randomly. Whole-mount in situ hybridizations were performed according to standard methods [30]. Gene-specific DIG-riboprobes were transcribed from pGEM-znf143a and pCI-myczSBF (zznf143b [16];) plasmids. The *znf143a* probe was generated using T7 RNA polymerase after linearization with *Not*I and hybridized only to the 3’UTR. The *znf143b* probe was generated using T3 RNA polymerase after linearization of pCI-myczSBF with *Ear*I. Therefore, the *znf143b* probe hybridized to the 3’UTR and a region of the ORF corresponding to the C-terminal 61 amino acids.

### CRISPRi

Guide RNAs from *Dra*I-linearized pDR274 templates were synthesized using the MAXIscript T7 in Vitro Transcription kit (Invitrogen) following an established protocol [28]. The concentrations of sgRNAs were determined using a Nanodrop spectrophotometer, and the quality of the RNA was verified by agarose gel electrophoresis. 70 ng of each sgRNA directed against a specific gene, and 3 μg of dCas9 protein (Integrated DNA Technologies; diluted to 1 mg/mL) were incubated at 37C for 10 min to form a mixture of dCas9:sgRNA complexes. Control samples contained dCas9 protein without sgRNAs. All injection samples were adjusted to a total volume of 10 μL using nuclease-free water and contained 0.2% phenol red. Approximately 1 nL of the dCas9:sgRNA mixture or dCas9 control was injected into one-cell zebrafish embryos. 30–60 embryos were injected with a single type of mixture per injection day.

## Data Availability

All data generated and analyzed during this study, and the materials constructed during this research, are available from the corresponding author on reasonable request.

## Abbreviations

3’UTR: 3′ Untranslated Region
cDNA: Complementary DNA
CRISPRi: CRISPR interference
CTCF: CCCTC-Binding Factor
DBD: DNA Binding Domain
dCas9: Deactivated Cas9
DIG: Digoxigenin
gRNA: Guide RNA
HEK293: Human Embryonic Kidney cell line
hpf: Hours post fertilization
mRNA: Messenger RNA
ORF: Open Reading Frame
qRT-PCR: Quantitative Reverse Transcriptase Polymerase Chain Reaction
SBF: SPH Binding Factor
SBS: Staf Binding Site
sgRNA: Single guide RNA
snRNA: Small nuclear RNA
SPH: *Sph*1 Postoctamer Homology
Staf: Selenocysteine transcription activating factor

## References

[1] Myslinski E, Gerard M-A, Krol A, Carbon P (2006). A genome scale location analysis of human Staf/ZNF143-binding sites suggests a widespread role for human Staf/ZNF143 in mammalian promoters. J Biol Chem.

[2] Ngondo-Mbongo RP, Myslinski E, Aster JC, Carbon P (2013). Modulation of gene expression via overlapping binding sites exerted by ZNF143, Notch1 and THAP11. Nucleic Acids Res.

[3] Myslinski E, Krol A, Carbon P (1992). Optimal tRNA^(Ser)Sec^ gene activity requires an upstream SPH motif. Nucleic Acids Res.

[4] Schuster C, Myslinski E, Krol A, Carbon P (1995). Staf, a novel zinc finger protein that activates the RNA polymerase III promoter of the selenocysteine tRNA gene. EMBO J.

[5] Kunkel GR, Cheung TC, Miyake JH, Urso O, McNamara-Schroeder KJ, Stumph WE (1996). Identification of a SPH element in the distal region of a human U6 small nuclear RNA gene promoter and characterization of the SPH binding factor in HeLa cell extracts. Gene Expr.

[6] Schuster C, Krol A, Carbon P (1998). Two distinct domains in Staf to selectively activate small nuclear RNA-type and mRNA promoters. Mol Cell Biol.

[7] Rincon JC, Engler SK, Hargrove BW, Kunkel GR (1998). Molecular cloning of a cDNA encoding human SPH-binding factor, a conserved protein that binds to the enhancer-like region of the U6 small nuclear RNA gene promoter. Nuc Acids Res.

[8] Bailey SD, Zhang X, Desai K, Aid M, Corradin O, Cowper-Sallari R, Akhtar-Zaida B, Scacheri PC, Haibe-Kains B, Lupien M (2015). ZNF143 provides sequence specificity to secure chromatin interactions at gene promoters. Nat Commun.

[9] Ye B-Y, Shen W-L, Wang D, Li P, Zhang Z, Shi M-L, Zhang Y, Zhang F-X, Zhao Z-H (2016). ZNF143 is involved in CTCF-mediated chromatin interactions by cooperation with Cohesin and other partners. Mol Biol.

[10] Mourad R, Cuvier O (2016). Computational identification of genomic features that influence 3D chromatin domain formation. PLOS Comp Biol.

[11] Myslinski E, Gerard M-A, Krol A, Carbon P (2007). Transcription of the human cell cycle regulated BUB1B gene requires hStaf/ZNF143. Nucleic Acids Res.

[12] Izumi H, Wakasugi T, Shimajiri S, Tanimoto A, Sasaguri Y, Kashiwagi E, Yasuniwa Y, Akiyama M, Han B, Wu Y (2010). Role of ZNF143 in tumor growth through transcriptional regulation of DNA replication and cell-cycle-associated genes. Cancer Sci.

[13] Michaud J, Praz V, Faresse NJ, JnBaptiste CK, Tyagi S, Schutz F, Herr W (2013). HCFC1 is a common component of active human CpG-island promoters and coincides with ZNF143, THAP11, YY1, and GABP transcription factor occupancy. Genome Res.

[14] Parker JB, Yin H, Vinckevicius A, Chakravarti D (2014). Host cell Factor-1 recruitment to E2F-bound and cell-cycle-control genes is mediated by THAP11 and ZNF143. Cell Rep.

[15] Paek AR, Lee C-H, You HJ (2014). A role of zinc-finger protein 143 for Cancer cell migration and invasion through ZEB1 and E-cadherin in Colon Cancer cells. Mol Carcinog.

[16] Halbig KM, Lekven AC, Kunkel GR (2012). The transcriptional activator ZNF143 is essential for normal development in zebrafish. BMC Mol Biol.

[17] Guschanski K, Warnefors M, Kaessmann H (2017). The evolution of duplicate gene expression in mammalian organs. Genome Res.

[18] Postlethwait JH, Woods IG, Ngo-Hazelett P, Yan YL, Kelly PD, Chu F, Huang H, Hill-Force A, Talbot WS (2000). Zebrafish comparative genomics and the origins of vertebrate chromosomes. Genome Res.

[19] Huang X, Miller W (1991). A time-efficient, linear-space local similarity algorithm. Adv App Math.

[20] Qi LS, Larson MH, Gilbert LA, Doudna JA, Weissman JS, Arkin AP, Lim WA (2013). Repurposing CRISPR as an RNA-guided platform for sequence-specific control of gene expression. Cell.

[21] Mandegar MA, Huebsch N, Frolov EB, Shin E, Truong A, Olvera MP, Chan AH, Miyaoka Y, Holmes K, Spencer CI (2016). CRISPR interference efficiently induces specific and reversible gene silencing in human iPSCs. Cell Stem Cell.

[22] Long L, Guo H, Yao D, Xiong K, Li Y, Liu P, Zhu Z, Liu D (2015). Regulation of transcriptionally active genes via the catalytically inactive Cas9 in C. elegans and D. rerio. Cell Res.

[23] Myslinski E, Krol A, Carbon P (1998). ZNF76 and ZNF143 are two human homologs of the transcriptional activator Staf. J Biol Chem.

[24] Ngondo-Mbongo RP, Carbon P (2013). Transcription factor abundance controlled by an auto-regulatory mechanism involving a transcription start site switch. Nucleic Acids Res.

[25] Conant GC, Wolfe KH (2008). Turning a hobby into a job: how duplicated genes find new functions. Nat Rev Genet.

[26] El-Brolosy MA, Stainier DYR (2017). Genetic compensation: a phenomenon in search of mechanisms. PLoS Genet.

[27] El-Brolosy MA, Kontarakis Z, Rossi A, Kuenne C, Gunther S, Fukuda N, Kikhi K, Boezio GLM, Takacs CM, Lai S-L (2019). Genetic compensation triggered by mutant mRNA degradation. Nature.

[28] Hwang WY, Fu Y, Reyon D, Gonzales APW, Joung JK, Yeh JR, MLe a (2015). Targeted Mutagenesis in Zebrafish Using CRISPR RNA-Guided Nucleases. In: CRISPR: Methods and Protocols, Methods in Molecular Biology.

[29] Kunkel GR, Tracy JA, Jalufka FL, Lekven AC (2018). CHD8short, a naturally-occurring truncated form of a chromatin remodeler lacking the helicase domain, is a potent transcriptional coregulator. Gene.

[30] Oxtoby E, Jowett T (1993). Cloning of the zebrafish krox-20 gene (krx-20) and its expression during hindbrain development. Nucleic Acids Res.

